# Vaccination against emerging and reemerging infectious diseases in places of detention: a global multistage scoping review

**DOI:** 10.3389/fpubh.2024.1323195

**Published:** 2024-01-29

**Authors:** Babak Moazen, Nasrul Ismail, Nisreen Agbaria, Sara Mazzilli, Davide Petri, Arianna Amaya, Jemima D’Arcy, Emma Plugge, Lara Tavoschi, Heino Stöver

**Affiliations:** ^1^Heidelberg Institute of Global Health, Heidelberg University, Heidelberg, Germany; ^2^Department of Health and Social Work, Institute of Addiction Research (ISFF), Frankfurt University of Applied Sciences, Frankfurt, Germany; ^3^School for Policy Studies, University of Bristol, Bristol, United Kingdom; ^4^Department of Translational Research and New Technologies in Medicine and Surgery, University of Pisa, Pisa, Italy; ^5^UK Health Security Agency, London, United Kingdom; ^6^Primary Care, Population Sciences and Medical Education, Faculty of Medicine, University of Southampton, Southampton, United Kingdom

**Keywords:** infectious diseases, vaccination, immunization, primary prevention, prisons

## Abstract

**Background:**

Despite the elevated risks of infection transmission, people in prisons frequently encounter significant barriers in accessing essential healthcare services in many countries. The present scoping review aimed to evaluate the state of availability and model of delivery of vaccination services within correctional facilities across the globe.

**Methods:**

Following the methodological framework for scoping reviews and adhering to the Preferred Reporting Items for Systematic Reviews and Meta-Analyses (PRISMA) extension for scoping reviews criteria, we conducted a systematic search across four peer-reviewed literature databases (Medline via PubMed, Web of Science, the Cochrane Library, Science Direct, and EBSCO), as well as 14 sources of grey literature. Two researchers meticulously examined the identified papers independently to extract pertinent data published between 2012 and 2022. The quality of the selected publications was assessed using established quality assessment tools.

**Results:**

Of the 11,281 identified papers 52 met the inclusion criteria. With the exception of one, all the included publications presented data from high-income countries, predominantly originating from the United States. Across the world, the most prevalent vaccines available in prison settings were COVID-19 and HBV vaccines, typically distributed in response to health crises such as pandemics, epidemics, and local outbreaks. Vaccine coverage and uptake rates within correctional facilities displayed noteworthy disparities among various countries and regions. Besides, individual and organizational barriers and facilitating factors of vaccination in prison settings emerged and discussed in the text.

**Discussion:**

The lack of vaccination services combined with low rates of vaccination coverage and uptake among people living and working in correctional facilities represents a cause for concern. Prisons are not isolated from the broader community, therefore, efforts to increase vaccine uptake among people who live and work in prisons will yield broader public health benefits.

## Introduction

Globally, over 11.5 million people are living in prisons and other places of detention on any day (1). People living in prisons (PLP) often lack access to adequate healthcare services in many countries (2). This situation not only represents a violation of their right to health but also contradicts international agreements such as the “the United Nations Standard Minimum Rules for the Treatment of Prisoners” commonly known as “the Nelson Mandela Rules” (3). The Nelson Mandela Rules clearly stipulate that “The provision of health care for prisoners is a State responsibility. Prisoners should enjoy the same standards of health care that are available in the community, and should have access to necessary health-care services free of charge without discrimination on the grounds of their legal status” (3). Yet, the lack of availability of healthcare services coupled with individual risk factors render PLP susceptible to various infectious diseases. This vulnerability is substantiated by the alarmingly elevated prevalence of infectious diseases among PLP, worldwide (4).

The recent COVID-19 pandemic has brought to light the sluggish and inadequate responses to controlling infection transmission in many prisons across the globe. Multiple past influenza outbreaks within prison facilities have resulted in numerous fatalities, underscoring the susceptibility of PLP to airborne diseases (5, 6). Moreover, since the outset of the COVID-19 pandemic, various stakeholders, including international organizations, prison healthcare professionals, scientists, and activists, had cautioned prison systems about the looming COVID-19 crisis on a global scale (7, 8). Nonetheless, the alarmingly elevated number of COVID-19 cases in prisons (9) serves as a glaring indicator of the inadequate response to the disease in numerous countries.

Although previous reviews have occasionally addressed vaccination in prison settings (10–13), there are still numerous aspects of vaccination in prisons that remain largely under-researched. This review is a part of the “Reaching the hard to reach: Increasing access and vaccine uptake among prison populations in Europe (RISE-Vac)” project co-funded by the European Union, aimed at enhancing the health status of people in Europe by increasing vaccine uptake among people who live and work in prisons in this region. Aligned with the aims and objectives of the RISE-Vac, the present review was conducted to map the following: (a) the availability, accessibility, and coverage of vaccination services, (b) models of vaccine delivery, and (c) to explore the perceived barriers and determinants of vaccine uptake and refusal in prisons.

## Methods

Methodology of the current review is published elsewhere extensively (14). Co-funded by the European Commission, the research initiative RISE-Vac aims to increase the rates of vaccine uptake within European prisons. Its objectives consist of identifying gaps in vaccine coverage, improving vaccine knowledge among PLPs and prison staff, and facilitating the transferability of the project’s health models and knowledge. Nine European partners from six countries participate in the RISE-Vac consortium: Germany, France, Italy, Moldova, Cyprus, and the United Kingdom. Detailed information about the project is available on the project’s website.^1^

### Data identification

This review adhered to the methodological framework for scoping reviews (15) and the PRISMA extension for scoping reviews (16). The data collection process comprised three key phases: first, a comprehensive literature search was conducted to explore both peer-reviewed and gray literature sources. Secondly, a public call for data was announced and disseminated through various platforms, including the Worldwide Prison Health Research and Engagement Network’s (WEPHREN) website, as well as social media channels, e.g., X (Twitter) and LinkedIn to access potential information not publicly available. Lastly, a system outreach was distributed via email among the international network of the authors as well as members of the RISE-Vac advisory board. The RISE-Vac advisory board comprises experienced researchers, prison health policymakers, healthcare providers, stakeholders affiliated with national, regional, and global organizations engaged in prison health, as well as experts who have personal experience of incarceration.

### Search strategy

Our search strategy was methodically executed across five distinct databases to identify peer-reviewed publications: Medline via PubMed, Web of Science, the Cochrane Library, Science Direct, and EBSCO. The goal was to procure insights into interventions geared toward increasing vaccine uptake within correctional facilities. In the pursuit of the optimal search query, a comprehensive exploration of Medical Subject Headings (MeSH), Entry terms, and non-MeSH keywords was undertaken. Subsequently, we settled upon the following search combination for our PubMed inquiry: ((Prison* OR Inmate OR Inmates OR Penitentiaries OR Penitentiary OR Jail OR Jails OR Detention Center OR incarcerat*) AND (Vaccin* OR Immunization)). The search terms were adapted for each database, given their unique search algorithms.

We expanded our search to scrutinize 14 gray literature sources, including WHO, CDC, ECDC, UNODC, WEPHREN, ResearchGate, Google, and Google Scholar. Tailored search terms were employed for each website to maximize precision. Particularly in Google, a wide array of terms, including vaccine-preventable diseases, were methodically combined with incarceration-related terms, with distinct combinations for each search.

### Inclusion/exclusion criteria and quality assessment

Although the initial database searches were conducted in English, publications in other languages were also identified and scrutinized. Inclusion criteria encompassed papers published in peer-reviewed scientific journals or gray literature between January 1, 2012, and December 31, 2022, reporting information on vaccination services for people who live and work in prisons. Conversely, papers published prior to 2012, those focused on pre-or post-incarceration periods, and those with no pertinent information were excluded. Our review imposed no restrictions regarding the age of the study participants, correctional setting types, or locations. Third reviewers (EP and LT) were consulted when discrepancies arose during the assessment. Additionally, for quality assessment we employed the National Institute of Health’s tools for quantitative research and the Critical Appraisal Skills Program checklist for qualitative research to evaluate the quality of the included papers.

### Data classification and analysis

We systematically extracted, categorized, and presented the key variables including publication year, location (country/region), scope, total prison population, sample size of study, publication type, type of setting, target population characteristics, model of delivery of the vaccination services (services are offered by who, where, and when), target diseases, and challenges encountered during implementation of the vaccination services in prisons. In this review, ‘coverage’ is used to denote the percentage of individuals who have received at least one dose of vaccine before their incarceration, while ‘uptake’ is used to indicate the percentage of individuals who have received at least one dose of vaccine while in detention centers.

## Results

### General characteristics of the included studies

**Figure 1:**
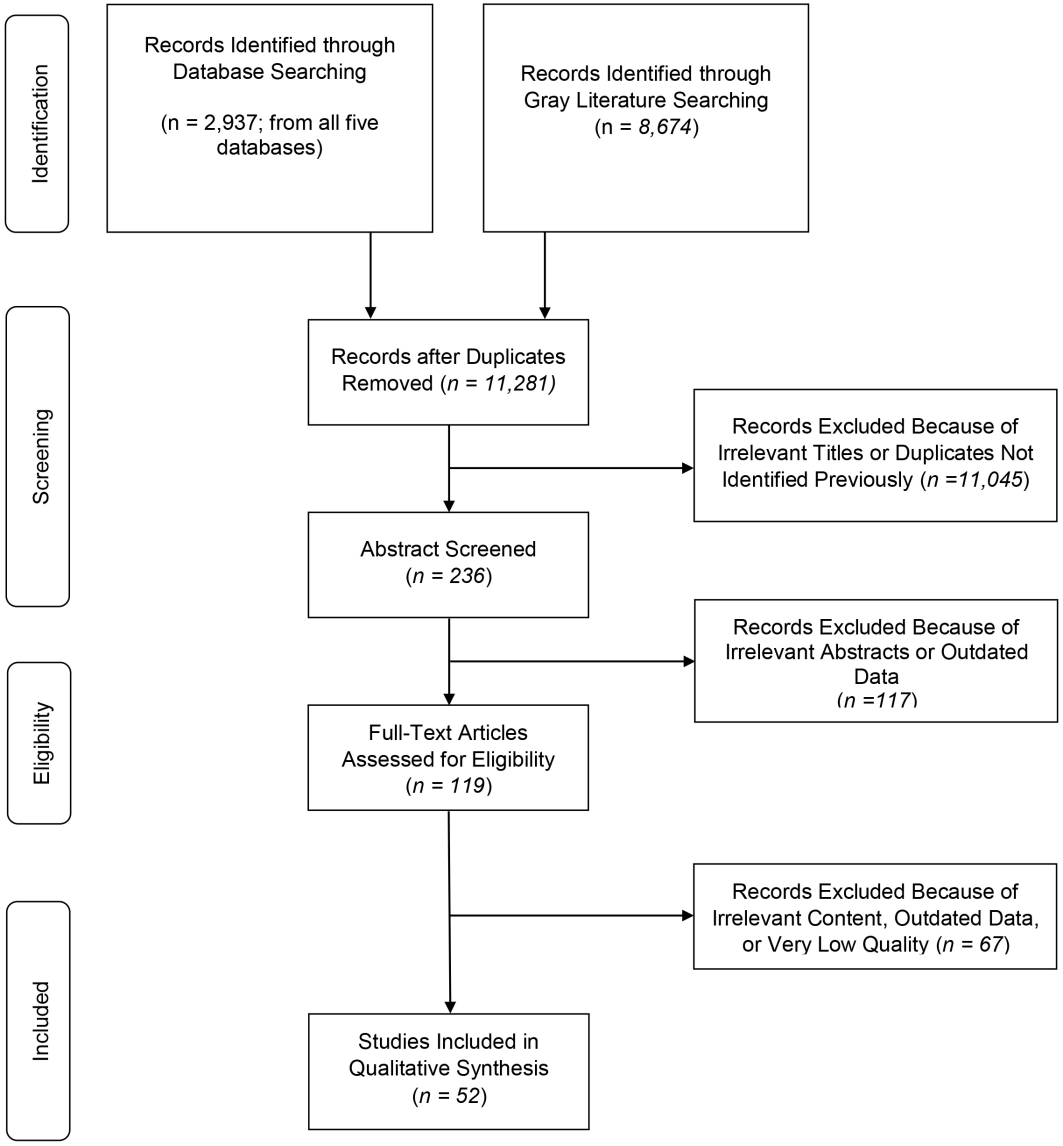
PRISMA chart of the included studies.

Of the 11,281 reviewed publications, 52 studies published between 2012 and 2022 met the inclusion criteria (Figure 1). The majority of the included publications were peer-reviewed in forms of original articles (35/52), followed by brief reports (10/52), research letters (4/52), opinion paper (1/52), case report (1/52), and research abstract (1/52). Most of the included publications came from high-income countries including the US (27/52), the UK (6/52), Canada (4/52), Italy (4/52), Australia (2/52), France (2/52), Switzerland (2/52), Spain (1/52), and Sweden (1/52). Aside from the high-income countries, we found data from only one middle income country: Thailand (1/52). Two included publications reported data at regional levels, both from Europe (one publication reporting data from seven countries, namely Spain, Northern Ireland, Ireland, Poland, Finland, Sweden, and the other from the EU/EEA countries). General characteristics of the included studies are presented in Table 1.

**Table 1:** Worldwide availability and model of vaccine delivery and uptake in prisons from 2012 to 2022.

Source/year of publication/Reference	Year of study	Country/Region	Scope	Type of setting(s)	Target population	Target disease/Vaccine	Doses delivered	Schedule completed	Coverage*	Uptake**	Determinants of/self-reported reasons for uptake	Determinants of refusal/Barriers to uptake	Comments
Allison et al., 2018 (17)	2016–17	US	3 Facilities in Kansas	Jail	PLP Adults B	HPV	NA	NA	NA	NA	Vaccines being offered at no cost	NR	Survey of intention
Allison et al., 2019 (18)	2017–18	US	1 Facility in Kansas	Jail	PLP Juvenile B 10–18 years	HPV	1	NR	NR	NR	Knowledge of vaccine and disease	Self-reported reasons to refuse: Side effects, confidentiality, pain from needle	Survey of intention plus vaccine offer
Beck et al., 2012 (19)	2003–10	England and Wales	147 Facilities (National)	Prison	PLP	HBV	NR	NR	22 (5–49%)	36% (16–59%)	NR	NR	
Berk et al., 2021(20)	2020–21	US	1 Facility	Prison with various facilities	PLP/STF	COVID-19	2	Yes (2 doses: booster after 4 months)	NR	First dose: 76.4% PLP 68.4% STF Booster: 77.7% PLP 69.6% STF	NR	NR	Response to an existing pandemic
Besney et al., 2017 (21)	2013	Canada	1 Facility	Remand facility	PLP from affected living units	Influenza	NR	NR	NR	PLP 95.5% (138/144)	NR	NR	Response to an existing outbreak; Only PLP on affected living units were offered vaccine
Biondi et al., 2022 (22)	2020–2021	US	National	All types	PLP	COVID-19	NR	Yes, in some institutions with a wide variation	NR	NR	Vaccine availability, preferences of PLP	Distrust in prison staff	Response to an existing outbreak
Borthwick et al., 2021 (23)	2017	UK	1 Facility	A high secure forensic mental health facility	Patients PLP	Influenza	1	Yes	NR	PLP 77.2%	Determinants of intention: past behavior, vaccine knowledge, cues to action Determinants of behavior: cues to action	NR	Vaccination For research purposes; Study of intention and behavior
Brinkley-Rubinstein et al., 2022 (24)	2021	US	6 Facilities	A jail-like intake facility	PLP/STF B	COVID-19	At least 1	NR	NR	NR	NR	NR	Response to an existing pandemic
Chatterji et al., 2014 (25)	2013	Australia	1 Facility	Correctional facility (no more detail)	PLP/STF B	Measles	NR	NR	NA	All except one PLP and two STF	NR	NR	Mass vaccination as a response to an outbreak
Chin 2021 (26)	2021	US	1 Facility	low-to-medium security prison	PLP	COVID-19	NR	Partly	56.6%	NR	NR	NR	Response to an existing pandemic
Costumbrado et al., 2012 (27)	2007–10	US	1 Facility	Jail	Self-defined MSM PLP	HAV/HBV	Up to four	Partly	NR	PLP: 1650 (42%) first doses; 1,215 (31%) second doses; 891 (23%) third doses; and 175 (4%) 12-month booster doses	those who had tested positive for any STI were more likely to start the immunization series	NR	MSM samples
Couper et al., 2013 (28)	2010–11	UK	1 Facility	Prison	PLP/STF	Influenza	NR	Partly	NR	STF: 20% PLP: NR	NR	Lack of audit of vaccine uptake due to the high turnover	Response to an existing outbreak
Da Costa et al., 2021 (29)	2021	Europe	Regional	Prison	PLP/STF	COVID-19	NR	Partly	Spain: Healthcare STF: 100% PLP: 97% Northern Ireland: PLP: 87.3% Poland: PLP: 74% Finland: PLP: 34.4% Ireland: PLP: 43.7% Sweden: PLP: 59.1%	NR	NR	NR	Response to an existing pandemic
Emerson et al., 2020 (30)	2016–17	US	1 Facility	Jail	Juveniles PLP (aged 9–18) and young adults (aged 19–26)	HPV	1	No	NR	No adults; 2 juveniles	Facilitator: A shared commitment to offering HPV vaccination services by leaders and staff in the two agencies	Barriers against collaboration between HD and jail: constrained resources and divergent organizational cultures and priorities Barriers to offer the vaccine: parental consent and the unpredictable, often brief duration of juvenile detent Ions; Potential barrier: criminal background check required by prison for “volunteers” (or non-employees) entering the jail	Study to find barriers and facilitators of collaboration between HD and jail to implement HPV vaccination; Vaccination offered for research purposes
Emerson et al., 2021 (31)	2017–18	US	4 states with 192 jails	Jail	NR	HPV	NR	NR	NR	1 jail has HPV program in place	Determinant of cooperation between HD and jail: Existing any vaccination program in jail	NR	Study to find determinants of cooperation between HD and jail to implement HPV vaccination
Fussilo et al., 2018 (32)	2017	Italy	1 Facility	Prison	PLP B	Measles	NR	NR	NR	First dose: 90 prisoners (74 males and 16 females); Second dose: 17 PLP	NR	NR	Vaccination for research purposes; After a month the program continued to vaccinate all PLP at entry
Gahrton et al., 2019 (33)	2017	Sweden	9 Facilities (1 county)	Prison	PLP B	HBV	1–3	Partly	NR	Full vaccine: 40.6% Susceptible to HBV and not received 3 doses of vaccine in prison combined with negative anti-HBs and negative anti-HBc: 18.6% Potentially susceptible to HBV and not received 3 doses in prison and not tested: 31%	NR	NR	
Gaskin et al., 2015 (34)	2011–12	US	1 Facility	Juvenile detention facility	PLP B	Tdap, MCV4, hepatitis A (HepA; two-shot series), varicella zoster virus (VZV; two-shot series), and HPV (Gardasil; three-shot series; offered routinely to boys and girls at the juvenile detention facility since 2009)	Various based on the type	Partly	Before prison vs. after prison: All 9 vaccines: 3% vs. 27%; Tdap: 63 vs. 91%; HAV 1st dose (76% vs. 92%); HAV 2nd dose: (58% vs. 79%); VZV 1st: 84 vs. 89% VZV 2nd: 47 vs. 65%; MCV4 1st dose: 51 vs. 85%; HPV 1st dose boys: 8% vs. 81%; HPV 1st dose girls: 38 vs. 85%; HPV 3rd dose boys (completed): 1 vs. 35%; HPV 3rd dose girls (completed): 18 vs. 45%	NR	NR	NR	Evaluating vaccination reports; Routine vaccination program exists in this facility
Getaz et al., 2016 (35)	2009	Switzerland	1 Facility	Pre-trial prison	PLP M serology-negative	HAV	NR	NR	NR	NR	NR	NR	Vaccines offered for research purposes
Di Giuseppe et al., 2022 (36)	2021	Italy	3 Facilities	Prison	PLP	COVID-19	NA	NA	NA	NA	Predictor: older age; Self-reported reasons to receive vaccine among those willing to uptake: safety, reduction of risk of infection, and effectiveness	Self-reported concerns among those unwilling to uptake: safety of vaccine, effectiveness of vaccine, is not recommended by physicians	KAP study on COVID-19 vaccine; Response to an existing pandemic
Goldman et al., 2022 (37)	2021	US	1 Facility	Juvenile detention center	PLP M (youths aged 10–21 years)	COVID-19	In total 50 doses	NR	97% unvaccinated 3% partially vaccinated	94% at least 1 dose 2% discharged	NR	Barriers to uptake: limited parental involvement to help access vaccination, feeling unlikely to be infected with COVID-19 or unlikely to become significantly ill, mistrust of the vaccines, and influence by adults who express mistrust, and misinformation about vaccine safety. Barriers to provide vaccine: lack of transportation, distance, and a need to provide advanced notice to probation officers	Response to an existing pandemic
Hagan et al., 2021 (38)	2020–21	US	National	Multiple settings under the coverage of the Federal Bureau of Prisons	PLP/STF B	COVID-19	1–2 doses (Janssen brand)	Partly	PLP 44.8% at least 1 dose 29.9% fully vaccinated	PLP: 0.3% were fully vaccinated before prison; Full uptake: 29.8%; STF: 50.2% at least 1 dose; 47.2% Full vaccination	Predictors of vaccine uptake: being male, being previously infected with COVID-19, higher age, and number of medical conditions associated with severe COVID-19	Predictors of vaccine refusal: being female, non-Hispanic black, Asian	Data from the Federal Bureau of Prisons in the US; Electronic registration system exists; Response to an existing pandemic
Hyatt et al., 2021 (39)	2021	US	National	Multiple settings	STF	COVID-19	NA	NA	NA	NA	Self-reported reasons for vaccine uptake: safety of the respondent and community, efficacy; Older, white and black participants reported being more likely to be vaccinated	Self-reported intention to refuse: 15.7–48.7%; Self-reported reasons of refusal: Being unsafe, no need when you are healthy; Young people and Ethno-racial groups including Hispanic, and American-Indian or Alaska Natives reported to be more likely to refuse vaccine	Study evaluating beliefs and self-reported reasons for vaccine refusal; Response to an existing pandemic
Jacomet et al., 2015 (40)	2012–13	France	1 Facility	Prison	PLP	HBV	NR	NR	NR	54.4%	NR	54.4% accepted and 12.1% refused to uptake. Due to the long delay for receiving serological test results and early release of PLP without consultation it was impossible to offer vaccines to the other PLP.	Vaccines were offered for research purposes.
Jeannot et al., 2016 (41)	2009–11	Switzerland	1 Facility	Juvenile correctional facility	PLP	Tdap Polio MMR HBV HPV	NR	NR	Tdap: 36.2% Polio 47.4% MMR 61.2% HBV 37% HPV 52.2% (Only females)	NR	NR	NR	Study on coverage of VPDs
Junghans et al., 2018 (42)	2016	UK	1 Facility	Prison	PLP	Measles	NR	Partly	NR	30%	NR	Barriers: delay in vaccine supply from the manufacturer, lack of staff, lack of protocols, rapid turnover; Reasons for refusal: Low trust in authorities, distrust of vaccine or vaccinator, and lack of knowledge	Mass vaccination at the time of outbreak
Khorasani et al., 2021 (43)	2013–20	US	1 State (14 facilities)	Jail	PLP	Influenza	1	partly	NR	1.9–11.8%	NR	Vaccine hesitancy, lack of a linkage system between the society and prison, and lack of a universal approach to influenza vaccination in the state	
Khorasani et al., 2021 (44)	2020–21	US	1 Facility	Jail	PLP/STF	COVID-19	NR	NR	NR	NR	NR	PLP: Determinants: Being black Self-reported reasons: Mistrust in vaccine Safety of vaccine Rushed timeline Effectiveness of vaccine STF: Predictor: Being health staff Self-reported: Concerns of safety efficacy Mistrust in vaccine Rushed timeline	Study of willingness Response to an existing crisis
CDC 2012 (45)	2010–11	US	1 Facility	Residential facility for children and youths	PLP with neurologic and neuro developmental conditions	Influenza	1 dose	Yes	NR	At least 10% (all 13 samples)	NR		Study at the time of crisis
Lessard et al., 2022 (46)	2021	Canada	3 Facilities	Prison	PLP B	COVID-19	NR	NR	NR	Self-reported desire to receive vaccine: 73%	Self-reported facilitators: environmental context and resources, social influences, beliefs about consequences, knowledge (reassurance about vaccine outcomes), and emotions (having experienced COVID-19-related stress)	Self-reported barriers: social influences (receiving strict recommendations, believing in conspiracies to harm), beliefs about consequences (infection control measures will not be fully lifted, concerns with vaccine-related side effects), and knowledge (lack of vaccine-specific information)	Qualitative Study of barriers and facilitators Study at the time of a pandemic
Leung et al., 2014 (47)	2020–11	US	1 Facility	Prison	PLP	Varicella	2 doses	Yes	NR	10 PLP out of 1,000 exposed	NR	NR	Vaccination at the time of outbreak
Li et al., 2020 (48)	2005–14	Australia	34 Facilities (National)	Prison	PLP (lifetime IDUs)	HBV	3 doses	Partly	NR	30%	NR	NR	HBV vaccines are available and offered to PWIDs in prisons in Australia
Liu et al., 2022 (49)	2021	US	1 State	Jail	PLP	COVID 19	At least 1	NR	NR	At least 1 dose: 56.2%	Older age, being woman, being vaccinated for influenza, living in shared housing	Concerns of side effects and efficacy, costs, need for an annual booster, mistrust of staff	Lower vaccine acceptance was observed in PLP than the general population; Study at the time of a pandemic
Moore et al., 2019 (50)	2016–17	US	1 Facility	NR	PLP F	HPV	NA	NA	NA	NA	NR	Self-reported barriers: Uncertainty about source of information, concerns about adverse reaction, mistrust of staff, and being gay or lesbian	A study of attitude in a facility that offers no vaccination
Moreau et al., 2016 (51)	2013	Canada	1 Facility	Youth offender correctional center	PLP M	Varicella	NA	NA	70% (single dose)	NA	NA	NA	Vaccination at the time of outbreak
Murphy et al., 2018 (52)	2016–17	US	3 Facilities	Prison	PLP	Varicella	1 or 2	Partly	NR	Prison 1: 48/384 (12.5%); Prison 2: 5/46 (10.9%); Prison 3: 7/97 (7.2%)	NR	NR	Vaccination at the time of outbreak
Nakitanda et al., 2021 (53)	2016–17	EU/EEA	Regional	prison	PLP	HBV	NR	NR	Coverage data from two countries: Estonia: 96 PLP Sweden: 66%	HBV vaccines available in 21/26 countries (80.8%); In 10 countries vaccines are offered to all eligible PLP; Czech Republic: offers vaccines for at risk groups; Sweden: only MSM; Netherlands: only upon request by physicians; Germany offers opt-out vaccine to all eligible PLP in 16 states, 5 to high-risk groups, opt-in (upon request) in 1 state	NR	NR	Regional data
Ortiz-Predes et al., 2022 (54)	2021	Canada	3 Facilities	Prison	PLP M	COVID-19	NR	NR	NR	NR	Self-reported reasons for acceptance: Education, incentives, receiving the vaccine from a trustworthy provider, vaccination of family and friends	Self-reported reasons for refusal: Low risk perception, universal distrust, STF attitudes and relationships, perceived unimportance of vaccines, negative past experience with vaccines, subjective norm, social pressure and social responsibility, role of media and communication, lack of info and accurate knowledge, religious and moral convictions, healthcare delivery, strict public health measures, and lack of incentives	Qualitative study of intention Study and vaccination at the time of pandemic
Parsons et al., 2021 (55)	2021	US	1 State	Prison	PLP	COVID-19	NR	No	NR	40% (still ongoing at the time of study)	NR	NR	Vaccination/study at the time of pandemic
Perret et al., 2013 (56)	2013	UK Wales	National	Prison	PLP/STF	HBV	NR	NR	NR	NR	NR	NR	Only mentioning the availability of HBV vaccination and interventions to increase access
Perret et al. 2019 (57)	2013–17	UK (Wales)	National	Prison	PLP M	HBV	1–3	Partly	1st dose from 2013 to 2017: 41.6% 50.3% 56.8% 56.8% 55.1% Full coverage: 28.7% 36.1% 37.8% 41% 39.6%	NR	NR	NR	
Perrodeau 2016 (58)	2013–14	France	1 Facility	Prison	PLP B	HBV	3	Partly	63% coverage of 2 doses for PLP who needed initial vaccination	NR	NR	NR	
Prince et al., 2022 (59)	2020–21	US	1 State	Prison	STF B	COVID-19	1–3	Partly	NR	First 2 months: 26% vs. 52% custodial vs. health STF By June 2021: 39% vs. 63% custodial vs. health STF	NR	Younger age, prior COVID-19 infection, residing in a community with relatively low rates of vaccination, sharing shifts with coworkers who had relatively low rates of vaccination	Study and vaccination at the time of pandemic
Ramaswamy et al., 2020 (60)	2017–18	US	4 States	Jail	PLP	HPV	NR	NR	2% of local health departments had HPV vaccine or planned to implement soon	NR	Parameters associated with interest in implementation: employees Perception of importance of vaccines, already providing a vaccine	Self-reported barriers to implement: Costs, PLP’s short length of stay, availability of medical STF	Survey of intention to implement vaccination
Ryckman et al., 2021 (61)	2020–21	US	1 State	Prison	PLP/STF	COVID-19	NR	Partly	36–76% PLP; 40% STF	NR	NR	NR	Vaccination and study at the time of pandemic; A modeling study
Sanchez et al., 2021 (62)	2018	US	1 Facility	Prison	PLP STF	Pneumonia	1	Partly	NR	78% PLP; 63% medical STF; 86% non-medical STF	NR	NR	Study and vaccination at the time of outbreak
Stasi et al., 2019 (63)	2016–17	Italy	1 Province (15/17 facilities)	Prison	PLP	HBV	1–3	Partly	NR	92.4% 1st dose; 83% 3rd dose	NR	Foreigners were significantly less likely to get vaccinated in prison	
Stasi et al., 2022 (64)	2016–17	Italy	1 Province	Prison	PLP	HBV	1–3	NR	NR	85.2% residents; 72% recently arrived	NR	NR	
Stern et al., 2021 (65)	2020	US	4 States	Prison and Jail	PLP	COVID-19	NA	NA	NA	NA	Predictors of willingness: Higher age, being in a prison rather than jail, being Hispanic/Latino (Hispanic) and American Indian/Alaska Native	NR	Study of willingness
Tiamkao et al., 2019 (66)	2014	Thailand	1 Facility	Prison	PLP	Diphtheria	NR	NR	NR	NR	NR	NR	Response to an existing outbreak
Vincente-Alcalde et al., 2020 (67)	2008–18	Spain	3 Facilities	Prison	PLP B	HAV HBV TD Pneumonia Influenza	NR	Partly	HBV: 52.3% vaccinated (75.7% completed schedule); HAV: 1.8% vaccinated (11.1% completed schedule); TD: 71.9% vaccinated (58.4% completed schedule); Pneumonia: 08% vaccinated/completed	Influenza: up to 16.2% between 2010 and 2013	Age was found to be a predictor	Problems: low quality of the records, poor and incomplete digitalization	Random selection of samples; Influenza vaccine was distributed during the study (no routine program)
Zellmer et al., 2021 (68)	2019	US	1 Facility	Jail	PLP B	HAV	NR	NR	NR	7.1% (that showed a significant increase from 0.6% after changing the protocols)	NR	NR	Response to an existing outbreak

PL, Peer-Reviewed Literature; GL, Grey Literature; M, Male; F, Female; B, Both Genders; NA, Not Available/Applicable; NR, Not Reported; RA, Review Article; BR, Brief Report; Abs, Abstract; LE, Letter to the Editor; STF, Staff; OA, Original Article; PLP, People Living in Prison and other closed settings; MM, Mixed-Methods; MSM, Men who have Sex with Men; PLWUD, People who Use Drugs; HD, Health Department; IDU, Injecting Drug User; TD, Tetanus-Diphtheria. *Of all subjects eligible for vaccination. **% of those eligible people receiving vaccine/booster doses in prison (if applicable).

### Quality of the included studies

Based on our assessment, none of the included studies were reporting high-quality evidence. Level of evidence was moderate-to-high in 3 publications, moderate in majority of the included publications (32/52), and low in 17 publications.

### Settings and samples

Sample size of the included studies varied widely from 46 to 164,283 participants. The included studies reported data from various settings including prisons (25/52), jails (10/52), various facilities combined (3/52), and other facilities, e.g., juvenile detention centers (14/52). Type of setting was not reported in one study. In the majority of the included publications, adult PLP were the main target population of the vaccination programs (36/52), followed by PLP and staff members combined (10/52), juvenile (3/52), and staff only (2/52). Target population was not reported in one publication. In 20 out of 52 publications, the gender of the target population was reported. Among these, the majority included both males and females (13 out of 20), while six publications focused solely on males, and one publication exclusively on females.

### Availability of vaccination services by country

**Figure 2:**
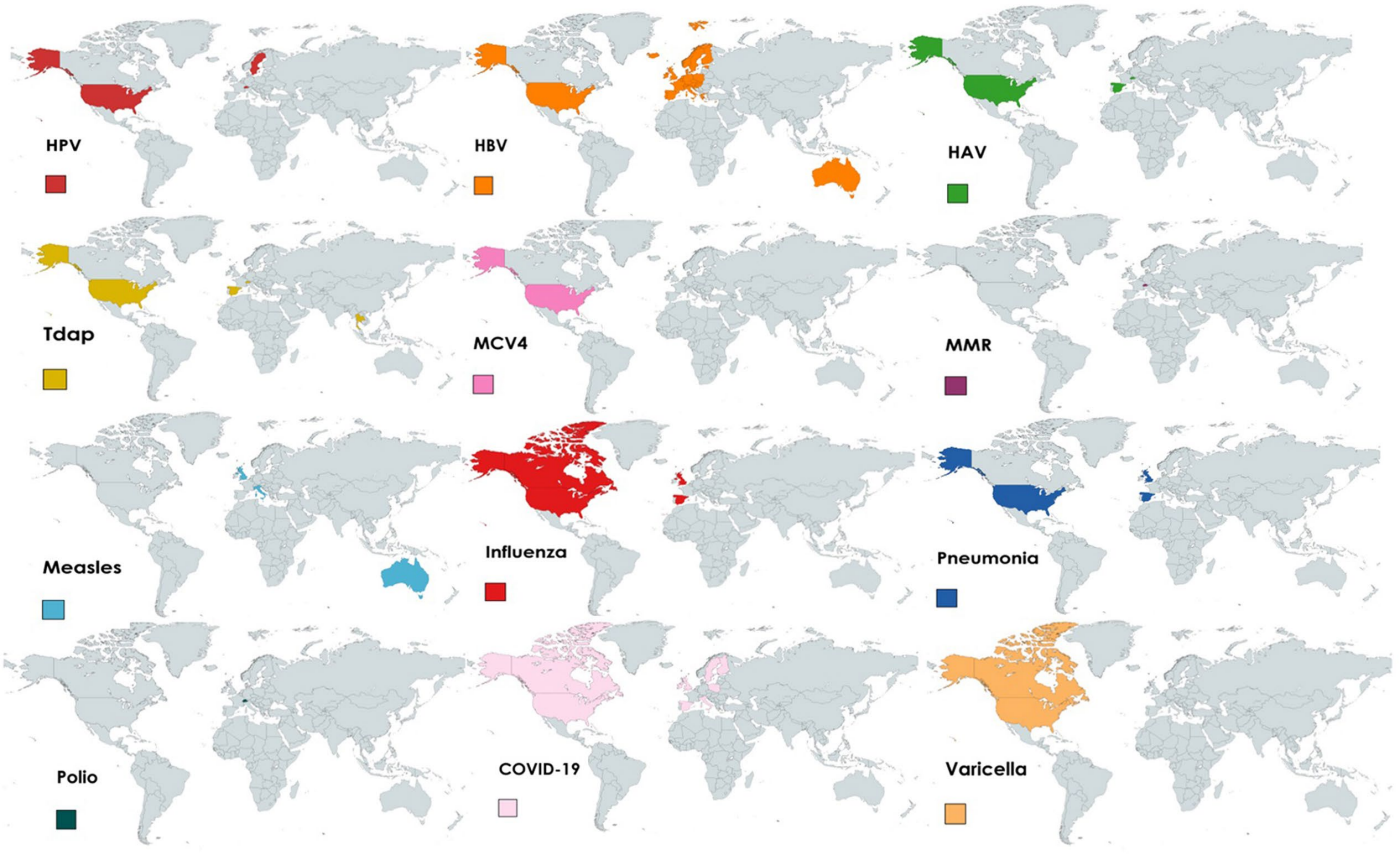
Availability of vaccines in prison settings by country, region, and type of vaccine from 2012 to 2022.

Included articles reported on vaccination programs in prisons covering various diseases including COVID-19 (17/52), HBV (10/52), HPV (6/52), influenza (5/52), measles (3/52), varicella (3/52), HAV (2/52), pneumonia (1/52), diphtheria (1/52), or two or more diseases combined (4/52). The countries implementing vaccination programs in prisons comprise Australia (measles, HBV), Canada (influenza, COVID-19, varicella), France (HBV), Italy (measles, COVID-19, HBV), Spain (HAV, HBV, Tdap, pneumonia, influenza), Sweden (HPV), Switzerland (HAV, Tdap, polio, MMR, HBV, HPV), Thailand (Tdap), the UK (HBV, influenza, measles), and the US (HPV, COVID-19, HAV, HBV, Tdap, MCV4, varicella, influenza, pneumonia). One of the papers reporting data at regional level reported that COVID-19 vaccines were available and offered in Spain, Northern Ireland, Poland, Finland, Ireland, and Sweden. According to the other regional publication, except in Bulgaria, Latvia, Lithuania and Romania, HBV vaccines were available in all EU/EEA countries. Figure 2 shows the availability of vaccines in prisons by country, region, and type of vaccine.

### Model of delivery of vaccination services in prisons

According to the included publications, in two European countries, Czech Republic and Sweden, HBV vaccines are offered only to at-risk populations (e.g., men who have sex with men (MSM)). In the Netherlands, HBV vaccines are offered only upon request by physicians. In Germany, nine out of the 16 states offered HBV vaccines to all eligible people, while five states offered the vaccines only to at-risk populations. HBV vaccines are available on an opt-in basis in one state in Germany.

Despite the lack of data, vaccines have reportedly been delivered in prisons by internal or external providers including clinical and non-clinical prison staff members, community healthcare workers, e.g., nurses and attending physicians, and researchers (in case the vaccination program was part of a research project). No study reported data on the time (e.g., immediately after admission or during incarceration) and location of delivery of vaccines in prisons.

In 25/52 included publications, the program was implemented as a response to existing health crises such as pandemics, epidemics, or local outbreaks. In one of the implemented interventions at the time of outbreak of influenza, only PLP in affected living units received the vaccines. In addition, evidence shows that in 6/52 settings offering vaccines there was no routine vaccination programs in place and vaccines were offered only for research purposes.

### Rates of vaccination coverage and uptake in prisons

Data on vaccination coverage among people who live and work in prisons were reported in 14 publications. Very low levels of coverage (0–25%) were reported from Spain, the UK, and the US; low levels (26–50%) from Finland, Ireland, Switzerland, the UK, and the US; moderate levels (51–75%) from Canada, France, Poland, Spain, Sweden, Switzerland, the UK, and the US; and high levels (76–100%) from Estonia, Northern Ireland, Spain, and the US (Table 2).

**Table 2:** Rates of vaccination coverage among PLP from 2012 to 2022^§^.

	COVID-19	HBV	HAV	HPV	VZV	MCV4	Tdap	MMR	Varicella	Polio	Pneumonia
US*	3-76		79	45	65	85	91				
Canada*									70		
Switzerland*		37		52.2			36.2	61.2		47.4	
Spain	97**	75.7*	11.1*				58.4*				8*
France*		63									
England-Wales***		22-41									
Northern Ireland**	87.3										
Ireland**	43.7										
Poland**	74										
Finland**	34.4										
Sweden**	59.1										
Estonia**		66									

^§^Data reported are expressed in percentages among PLP; data on HAV and HBV refer to the complete course of vaccination. *Data reported from one to three facilities.**Regional data.***National data.

Data on vaccine uptake among people who live and work in prisons were reported in 28 included publications. Very low levels of uptake (0–25%) were reported from Italy, Spain, Sweden, the UK, and the US; low levels (26–50%) from Australia, Sweden, the UK, and the US; moderate levels (51–75%) from France, Italy, the UK, and the US; and high levels (76–100%) from Canada, Italy, the UK, and the US (Table 3).

**Table 3:** Rates of vaccination uptake among PLP from 2012 to 2022^§^.

	COVID-19	Influenza	HBV	HAV	MMR	Varicella	Pneumonia
US*	29.8–94	1.9–11.8	23	7.1–23		7.2–12.5	78
Canada*		95.5					
Spain*		16.2					
France*			54.4				
England-Wales		20–77.2*	36***		30*		
Italy			83–85.2**		3.9*		
Sweden**			40.6				
Australia***			30				

^§^Data reported are expressed in percentages among PLP; data on HAV and HBV refer to the complete course of vaccination. *Data reported from one to three facilities. **Regional data. ***National data.

### Factors facilitating vaccine delivery and uptake in prisons

At an individual level, higher levels of education, knowledge of vaccine and disease, vaccination being offered free of charge, recommendation from trusted individuals, history of vaccination, history of contracting infectious diseases, gender, living in shared housing, the offer of incentives to get vaccinated, immunization status of family members and friends, race, and cues to action were reported to be the facilitators of vaccine uptake among PLP. For staff members, older age, race (white and black), belief in safety and efficacy of vaccines, and protection of the community were found to be the facilitators of vaccine uptake. At an organizational level, availability of vaccines and type of facility (living in a prison rather than other facilities) were reportedly facilitators of vaccine uptake.

### Barriers toward vaccine delivery and uptake in prisons

Barriers toward vaccine uptake in adult PLP at an individual level included cost of vaccines, concerns of safety and efficacy, concerns of confidentiality, pain from needle, distrust in prison staff members, recommendation from people other than physicians, female gender, race (non-Hispanic black, Asian, American-Indian, and Alaska native), social pressure and social responsibility, religious and moral convictions, being foreign national, and lack of incentive. For juvenile PLP, limited parental involvement to increase vaccine uptake, distrust in prison staff members, being influenced by adults who express mistrust, need to secure parental consent, low perceived risk, and often brief duration of detention were reportedly the main barriers to vaccine uptake. Among staff members, younger age, history of infection, living in a community with low rates of vaccination, sharing shifts with coworkers with low rates of vaccination, being a healthcare worker, concerns of safety and efficacy of vaccine, and rushed timelines were found to be the barriers toward vaccine uptake.

At an organizational level, high turnover of PLP, long delay in receiving serological results and release of PLP, delay in vaccine supply by the manufacturer, strict public health measures, shortage of staff members, lack of protocols, lack of transportation, distance, and necessity to provide advanced notice to probation officers were reportedly the barriers toward vaccination offer and uptake in prisons, worldwide.

## Discussion

Our review revealed that evidence regarding the availability of vaccination services in prisons primarily originates from high-income countries. However, data on the accessibility, acceptability, quality, and the delivery models of vaccination services in prison settings remain limited. Vaccine coverage and uptake rates within prisons exhibit significant variations across different countries and regions. COVID-19 vaccination stood out as the most frequently reported vaccine in prisons, underscoring the lack of attention given to other vaccine-preventable diseases within correctional facilities worldwide. Due to the paucity of data, the coverage and uptake of vaccines in prisons exhibit substantial disparities depending on the country and the specific type of vaccine. Notably, many of the included publications indicated that vaccination services were typically implemented during times of crises such as pandemics, epidemics, and local disease outbreaks. This highlights the absence of routine vaccination programs within the prison systems across the globe. Moreover, our investigation identified various individual and organizational barriers that hinder the provision and uptake of vaccines within prison settings worldwide.

The findings of this review align with previous studies (10, 11) and underscore the limited availability of vaccines in prisons and the low vaccination coverage among PLP. Our review has highlighted that certain countries primarily offer vaccination services during crises such as epidemics and pandemics. Furthermore, it is important to note that in some cases, not all people living in a prison receive vaccines, but rather, only those in affected living units (21). Vaccines are recognized as one of the most effective preventive measures for reducing the incidence, morbidity, hospitalization, and mortality associated with infectious diseases within correctional facilities (69). However, relying solely on vaccination as a responsive strategy during crises could undermine its overall effectiveness in mitigating the health and financial burdens of infectious diseases in prison settings.

Having knowledge about vaccines and infectious diseases was identified as a significant facilitator for vaccine uptake among incarcerated people. On the other side, misinformation and disinformation are among the major factors hindering vaccine uptake, as observed widely during the COVID-19 pandemic across the world. In this regard numerous studies have shown the negative association between vaccine hesitancy and level of knowledge as well as between vaccine hesitancy and behavioral intention (70, 71). As social contacts are one of the most common sources of health information among PLP (72), the risk of dissemination of misleading information about healthcare services, e.g., vaccination is high. Some community-based recommendations, e.g., active participation of healthcare professionals to address misleading information (73) can be adapted and implemented in prison settings as well. This should be taken into consideration that immediate response plays a crucial role in tackling infodemics (74).

Vaccine hesitancy, however, is multifaceted and goes beyond misleading information alone. Various multicomponent dialog-based interventions have been recommended to address vaccine hesitancy in the community (75). These recommendations include but not limited to targeting specific populations, e.g., unvaccinated or under-vaccinated; increasing knowledge and awareness on vaccines; enhancing access and convenience of vaccination services; to engage influencing people in the program; embedding vaccination services in routine healthcare practices and procedures; and addressing mistrust in healthcare providers and institutions through engagement and dialog (75). While these recommendations are primarily designed for the general population, they can also be applied in correctional settings to enhance vaccine uptake among people who live and work within prisons.

Various interventions have been implemented to address vaccine hesitancy and to increase vaccine uptake in prison settings around the world. These interventions are mostly focused on information dissemination through educational interventions including courses with or without panel discussions, posters, factsheets, pamphlets, etc. (14). In addition to the educational interventions, some countries have implemented organizational interventions including implementing the vaccination programs by external healthcare providers, applying accelerated vaccination schedules for hepatitis B, modifying the vaccination protocols to offer vaccines at the time of entrance, and prioritizing PLP for vaccination implemented by the governments. As evidence on the effectiveness of the aforementioned interventions is scarce (14), these interventions should be implemented cautiously.

Overcrowding stands as one of the most pervasive issues and a significant contributor to substandard prison conditions on a global scale, which, in turn, significantly compromises the quality of healthcare services within correctional facilities. Evidence shows that prisons in 118 countries currently exceed their maximum occupancy limits (76). In the United States, for instance, overcrowding has resulted in inmates having to sleep in gyms, hallways, and even triple-and quadruple-bunked in their cells (77). Numerous strategies, as recommended in the literature, can be employed to mitigate overcrowding within prison settings. These strategies include diverting minor cases away from the criminal justice system; enhancing access to justice and improving case management during pre-trial detention; fostering the development and implementation of constructive non-custodial measures and sentences; reducing sentence lengths while ensuring a consistent approach to sentencing; and establishing avenues for parole or other forms of early release, along with comprehensive post-release support to deter recidivism (78). Applying these measures can effectively alleviate overcrowding and, consequently, enhance the quality of healthcare services, including the administration of vaccinations, in prisons worldwide.

The recently-published WHO framework for assessing prison health system performance has been developed to assist countries in enhancing their prison health systems (79). To achieve this objective, the framework outlines five key priorities: strengthening prison information systems to improve surveillance and response capacity; monitoring health service provision within correctional facilities; tracking and evaluating system performance; acquiring valid and reliable measures of the health status of incarcerated individuals; and engaging in intersectoral collaboration to enhance overall performance and outcomes. Incorporating these components into the design and implementation of healthcare services, including vaccination programs, will significantly enhance the quality and sustainability of these services.

People who work in prisons can play a significant role in bringing the infectious diseases, in specific airborne diseases, from the community to prisons and vice versa. Yet, the health assessment of people who work in prisons has historically been overlooked. During the COVID-19 pandemic, for example, low vaccination rates among prison staff members were reported from many countries (59, 80). Evidence also shows that in many countries implementing COVID-19 vaccination programs prison staff members were not among the priority groups to receive the vaccine (81). Besides that, in our review we found 10/52 publications including both people who live and work in prisons and only 2/52 publications targeting prison staff alone. The lack of publication is another factor highlighting the lack of attention to prison staff members as a key population in prisons. It should be considered that prison-based vaccination plans excluding people who work in prisons would be incomplete and suboptimal.

Task shifting entails the purposeful redistribution of tasks to healthcare providers with fewer qualifications, extending beyond their traditional scope of work (82). When supported by strong evidence and executed efficiently, task shifting can significantly enhance health outcomes and contribute to the long-term sustainability of healthcare systems (83). In particular, task shifting has demonstrated its effectiveness and viability in managing infectious diseases (84, 85). Given that a shortage of human resources is a primary obstacle to delivering optimal healthcare services within correctional facilities, the adoption of task shifting, involving non-medical staff members in healthcare service delivery, is anticipated to offer a solution for enhancing the quality and long-term sustainability of healthcare services including vaccination in prison settings.

### Strengths and limitations of the review

This review is, to our knowledge, the first of its kind classifying and reporting on the characteristics of the existing vaccination programs in prisons in the world. However, results of the present review should be seen in light of some limitations. Lack of published data on various aspects of vaccination in prisons is one of the main limitations of the current review and, on a broader scale, one of the most important barriers toward taking evidence-based decisions on prison health globally. The neglected aspects of vaccination in prisons comprise gender and racial disparities in vaccine uptake and hesitancy; subpopulations of PLP, e.g., the LGBTQ+, older adults, and those living with chronic conditions; determinants of vaccine uptake and refusal; and strategies to increase vaccine uptake in places of correction. Lack of quality was another main limitation of this review, as none of the included studies were found to be reporting high-quality evidence. In our review we addressed these limitations using a multistage search strategy, applying a wide range of inclusion/exclusion criteria, and taking advantage of the established quality assessment tools.

### Recommendations and future directions

We propose the following recommendations are made to enhance the quality of vaccination services in prison settings, to address vaccine hesitancy, and to increase the rates of vaccine uptake among people who live and work in prisons:

- To establish more valid and reliable data that can inform prison policy-makers and enhance the effectiveness and quality of the vaccination services in prison settings, funding organizations should expand their support on prison health research, and prison policy-makers should facilitate data collection in prison settings.
- To address mistrust and distrust of vaccination among prison staff members, health providers should take forward the implementation of vaccination services and related interventions, e.g., knowledge dissemination.
- To fight against infodemics, policymakers should capitalize the knowledge of lived experience to provide PLP with reliable and updated information through peer-led educational activities and ensure they are able to make an informed decision on vaccination.
- Implementing mandatory interventions including vaccination, or putting sanctions/restrictions for not using services, is violation of the rights of PLP as human beings; therefore, use of the entire healthcare services in prison settings must be voluntarily with no obligation. At the same time, evidence-based strategies should be in place to facilitate access to services and increase service uptake.
- Policymakers should undertake needs assessment to identify the needs of populations and subpopulations of PLP before implementing vaccination services. All interventions should be tailored based on the needs of the target populations considering their age, gender, race, sexual orientation, and cultural diversity.
- Monitoring and evaluation should be in place to track the effectiveness of the implemented vaccination interventions in prisons. Routine monitoring and evaluation will help prison policymakers and healthcare providers identify the gaps and find solutions to address the possible problems.
- High turnover and short duration of stay is one of the contributing factors preventing PLP from finishing vaccination schedules of vaccines requiring more than one dose. Therefore, there is a need for a referral system to ensure completion of vaccine schedules among people with unfinished vaccination schedules after release.
- The immunization status of those who work in prisons should be checked before and during their employment on a regular basis.

## Conclusion

In this review we evaluated over 11,000 publications and found that very few countries, worldwide, offer vaccines to people who live and work in prisons. The most frequently-offered vaccine in prison settings was COVID-19, underlining the lack of attention to the other vaccine-preventable diseases in prisons during the past decade. Similarly, the vast majority of the included publications came from high-income countries and regions, highlighting the abandonment of prison health in low-and middle-income settings. It should be considered that over 90% of PLP will eventually return to their communities. On the other side, prison staff members commute daily between prisons and communities. Therefore, providing accessible, acceptable, affordable and high-quality vaccination services for people who live and work in prisons is a public health investment. Apart from their public health aspects, provision of healthcare services in prison settings is an effort toward reaching international targets such as the UN sustainable development goal 3 of ensuring healthy lives and promoting well-being for all.

## Data availability statement

The original contributions presented in the study are included in the article/supplementary material, further inquiries can be directed to the corresponding author.

## Author contributions

BM: Conceptualization, Investigation, Methodology, Software, Supervision, Visualization, Writing – original draft, Writing – review & editing. NI: Conceptualization, Investigation, Methodology, Writing – review & editing. NA: Conceptualization, Investigation, Methodology, Writing – review & editing. SM: Conceptualization, Investigation, Methodology, Writing – review & editing. DP: Conceptualization, Investigation, Visualization, Writing – review & editing. AA: Conceptualization, Investigation, Validation, Visualization, Writing – review & editing. JD'A: Conceptualization, Investigation, Methodology, Validation, Writing – review & editing. EP: Conceptualization, Investigation, Methodology, Supervision, Validation, Writing – review & editing. LT: Conceptualization, Funding acquisition, Investigation, Methodology, Project administration, Supervision, Writing – review & editing. HS: Conceptualization, Investigation, Methodology, Project administration, Supervision, Validation, Writing – review & editing.

## Footnotes

^1^https://wephren.tghn.org/rise-vac/
